# Occurrence of Ergot Alkaloids in Barley and Wheat from Algeria

**DOI:** 10.3390/toxins13050316

**Published:** 2021-04-28

**Authors:** Laura Carbonell-Rozas, Choukri Khelifa Mahdjoubi, Natalia Arroyo-Manzanares, Ana M. García-Campaña, Laura Gámiz-Gracia

**Affiliations:** 1 Department of Analytical Chemistry, Faculty of Sciences, University of Granada, 18071 Granada, Spain; rozas@ugr.es (L.C.-R.); choukri_khelifa@hotmail.com (C.K.M.); amgarcia@ugr.es (A.M.G.-C.); 2 Department of Biology, Faculty of Natural and Life Science, University of Oran, Oran 31100, Algeria; 3 Department of Analytical Chemistry, Faculty of Chemistry, University of Murcia, 30100 Murcia, Spain; natalia.arroyo@um.es

**Keywords:** ergot alkaloids, cereals, ultra-high performance liquid chromatography-tandem mass spectrometry, exposure assessment, Algeria

## Abstract

The natural occurrence of six major ergot alkaloids, ergometrine, ergosine, ergotamine, ergocornine, ergokryptine and ergocristine, as well as their corresponding epimers, were investigated in 60 cereal samples (barley and wheat) from Algeria. Ultra-high performance liquid chromatography coupled to tandem mass spectrometry (UHPLC-MS/MS) and a QuEChERS extraction method were used for sample analysis. The results revealed that 12 out of 60 samples (20%) were contaminated with ergot alkaloids. Wheat was the most contaminated matrix, with an incidence of 26.7% (8 out of 30 samples). The concentration of total ergot alkaloids ranged from 17.8 to 53.9 µg/kg for barley and from 3.66 to 76.0 μg/kg for wheat samples. Ergosine, ergokryptine and ergocristine showed the highest incidences in wheat, while ergometrine was the most common ergot in barley.

## 1. Introduction

Ergot alkaloids (EAs) are mycotoxins produced by different fungi of the *Clavicipitaceae* family, such as *Claviceps purpurea*, *Claviceps paspali* and *Claviceps fusiformis*, which are prevalent in cereals such as rye, wheat, barley, millet, triticale and oat [1]. The fungus produces distinctive black sclerotia that contain variable amounts of EAs (0.01–0.50%) [2,3]. The EA pattern and contents in sclerotia vary with the individual fungal strain, geographical region and the host plant. Therefore, there are multiple factors that affect EA production, and among the important ones are the weather conditions, as EA production is favored by heavy rainfall and wet soils [4].

Sclerotia are collected together with cereals, so they can cause contamination of cereal-based foods with EA, and their ingestion can cause ergotism, a mycotoxicosis known since ancient times. In the Mediaeval period, this disease was known as St. Anthony’s Fire or Holy Fire, due to the intense pain resulting from vasoconstriction and subsequent gangrene and the neurotoxic symptoms associated with the ingestion of EAs [5]. Nowadays, ergotism has been almost eradicated as a human disease, although some cases have been reported recently in France, India and Ethiopia [2,6,7]. Moreover, it remains an important veterinary issue [2,8]. Indeed, an increase of EA contamination in cereals and cereal-based products is being observed due to new cereal hybrids susceptible to *C. purpurea* [9] and climate changes [10].

The most common EAs produced by *C. purpurea* are ergometrine (Em), ergosine (Es), ergotamine (Et), ergocornine (Eco), ergokryptine (Ekr) and ergocristine (Ecr) [2,8]. These molecules are amide-like derivatives of lysergic acid, with a double bond at C9−C10 of the ergoline ring and an *R* configuration at C8, indicated by the suffix “-ine” in their names, which undergo reversible epimerization to C8*(S)*, indicated by the suffix “-inine”, that is: ergometrinine (Emn), ergosinine (Esn), ergotaminine (Etn), ergocorninine (Econ), ergokriptinine (Ekrn) and ergocristinine (Ecrn). The epimerization process is rapid and not yet well understood, and to avoid underestimation of the total EA content, both forms (*R* and *S*) must be quantified [11]. The chemical structure of EAs is shown in the Supplementary Materials (Figure S1).

Limits for individual EAs in cereals have not yet been set (although they are likely to be fixed in the near future), and in the European Union (EU), the presence of ergot sclerotia is regulated to a maximum of 500 mg/kg in unprocessed cereal (with the exception of corn and rice) for humans [12] and 1000 mg/kg in feed materials and compound feed containing unground cereals [13]. Moreover, a correlation between the sclerotia content and EA concentration in grains has been reported [14].

In 2012, upon a European Commission request, the Panel on Contaminants in the Food Chain (CONTAM Panel) [2] delivered a scientific opinion on the risks to human health related to the presence of EAs in food and feed, where they derived a group acute reference dose (ARfD) of 1 µg/kg body weight (bw) and a tolerable daily intake (TDI) of 0.6 µg/kg bw/day for the sum of the six principal alkaloids, (-ine forms) as well as their corresponding epimers (-inine forms). These health-based guidance values (HBGVs) are based on the conclusions of the CONTAM Panel, which considered the vasoconstrictive effect on tail muscular atrophy in rats as the critical effect for hazard characterization, as also in humans the vasoconstriction in the limbs is the most critical effect, causing dry gangrene [2].

Different methods have been reported for the analysis of EAs in cereals. The most widely used are based on liquid chromatography (LC) coupled with different detection techniques, such as fluorescence [15,16] and, most frequently, tandem mass spectrometry (MS/MS). Moreover, a sample treatment is required before determination to remove interferences and pre-concentrate the analytes. These treatments are usually based on a solid-liquid extraction followed by liquid–liquid extraction (LLE) or solid phase extraction (SPE) for cleaning-up [16,17,18]. Several reviews have covered the different methods for the determination of EAs in foodstuff [17,19].

In Algeria, wheat and barley are important and highly consumed cereals in the diet. The importance of these cereals lies in their easy transformation into flour and semolina, which are the main ingredients for “homemade” muesli, bread, pasta and other bakery products. However, these cereals can be a source of EA contamination. According to some authors [3], the occurrence of EAs in the cleaned cereals come from the presence of very small fragments, or dust, of sclerotia adhering to the grains, since the physical cleaning practices cannot eliminate all. Additionally, dry climatic conditions (typical in Algeria) favored the development of sclerotia similar in size to grain, making their separation difficult [4]. Moreover, in Algeria, there is no maximum level for mycotoxins, and knowledge about their contamination levels is scarce, especially regarding EAs [20,21]. Therefore, Algerian consumers of these cereals could be exposed to EAs. Thus, quality control of those cereals throughout the entire food chain would be a key part of ensuring food safety.

Within this context, the aims of this study were: (i) to develop and validate a selective UHPLC–MS/MS method for the quantification of the six principal EAs (-ine forms: Em, Es, Et, Eco, Ekr and Ecr) as well as their corresponding epimers (-inine forms: Emn, Esn, Etn, Econ, Ekrn and Ecrn) in barley and wheat; (ii) to evaluate the levels of these mycotoxins in 60 samples of barley and wheat commercialized in Algeria; (iii) to make an initial evaluation of the daily intake of EAs through barley and wheat consumption for the Algerian population.

## 2. Results and Discussion

### 2.1. Calibration and Performance Characteristics of the Method

Table 1 summarizes the results for procedural calibration, limits of detection (LODs) and limits of quantification (LOQs) for barley and wheat. All EAs exhibited a good linearity over the working range in all the cases (determination coefficients R^2^ > 0.99). The LOQs ranged between 0.50–3.92 μg/kg for barley and 0.49–3.33 μg/kg for wheat.

As can be seen in Table 2, the matrix effect was lower than 16% (absolute value) for all EAs, except for Em and Emn, which showed a significant signal suppression in both matrices. As a consequence, procedural calibration curves were used for quantification. Trueness (expressed as recoveries and summarized in Table 2) were within 86.6–105% for barley and 84.9–109% for wheat. Results of precision study (intra-day and inter-day precision) are shown in Table 3, and were lower than 11% in all the cases. The obtained values were considered satisfactory under the recommendations for determination of contaminants [22].

### 2.2. Occurrence of Ergot Alkaloids

The validated method was applied to monitor the natural occurrence of EAs in barley (n = 30) and wheat (n = 30) samples intended for human consumption in Algeria. Each sample was analyzed in triplicate. For identification of the analytes, SANTE guidelines recommendations were followed [23]. Detailed results for positive samples (including concentrations for individual EAs) are available in the Supplementary Materials (Table S1), while the summary of the results can be seen in Table 4.

Of the 60 analyzed samples, 12 samples (20%) were found to be positive for EAs. Overall, wheat was the most contaminated matrix, with an incidence of 26.7% (8 out of 30 samples), with concentrations ranging from 3.66 to 76.0 μg/kg (total content of EAs, considering only concentrations higher than LOQ) and a mean concentration of 33.1 μg/kg. On the other hand, in barley, 4 out of 30 analyzed samples (13.3%) were found to be contaminated with EAs at concentrations ranging from 17.8 to 53.9 μg/kg (total content of EAs, considering only concentrations higher than LOQ), with a mean concentration of 35.4 μg/kg.

These results are globally in line with some recent studies on the occurrence of EAs in cereals. Thus, the study developed by the EFSA collecting data from 2011 to 2015 from 15 different European countries revealed that in 76% cereal-based samples EAs were unquantified [24]. Other surveys revealed the presence of several EAs in barley and wheat samples from Norway, but concentrations were generally low [25]. Additionally, an occurrence study carried out during three years reported low incidence of total EAs in French durum wheat (15–30%), wheat (23–30%) and barley (27–39%) [18], and the analysis of 123 Chinese cereal samples revealed that only five samples were contaminated with EAs at a concentration range of 1.01–593 μg/kg [26]. Nevertheless, other studies reported higher incidences of EAs: for instance, EAs were found in 54% of 113 grain-based products for infant and young children from the Netherland, with mean levels of 10.6, 6.2 and 8.6 µg/kg for 2011, 2012 and 2013, respectively [3]; in Italy, 62 out of 71 samples of wheat and rye were contaminated with at least one EA, with total EA concentrations similar to those reported in our study, although one sample showed a concentration up to 1142.6 μg/kg [27]. In addition, EAs were detected in 10 of 13 wheat samples from different European countries, with total EA concentrations up to 7654 μg/kg [28]. Furthermore, 104 out of 122 samples of cereals-based food and feed from Belgium were positives (concentrations ranging from 1 to 1145 µg/kg) [1]; and 23 out of 39 grain samples from Luxemburg were contaminated with EAs with concentrations from 0.7 to 2530.1 μg/kg, even after sieving to remove sclerotia [16]. To the best of our knowledge, there are no reports about the occurrence of EAs in African countries.

The occurrence of EAs among the analyzed samples showed some differences from one region to another; thus, 100% and 37.5% positive samples of barley and wheat, respectively, were from Tiaret, 50% positives samples of wheat were from Oran and only 12.5% positive samples (one out of eight samples) of wheat were from Aint Temouchent. These results could be a consequence of the influence of climatic and geographical conditions on the occurrence of EAs in cereals, as Oran and Aint Temouchent are close to the coast and have a dry Mediterranean climate, while Tiaret is located inland. However, these are only tentative conclusions, as no information about the cereal growing place was available, and could be different from those locations. In this sense, it would be interesting to collect more data about the incidence of EAs in these cereals in different years and locations from Algeria, in order to properly study the influence of climate throughout the country.

### 2.3. Distribution of Individual Ergot Alkaloids

Among the positive samples, and considering the detected EA (concentrations higher than LOD), the distribution of individual EAs varied. Table 5 presents the incidence of the individual EAs in the analyzed samples. Globally, Em was the most frequent EA (detected in six wheat and four barley samples). Considering only concentrations higher than LOQ, Es, Ekr and Ecr in wheat and Em in barley were the most frequent EAs. The highest concentrations of an individual EA were 28.6 µg/kg for Ecr and 50.0 µg/kg for Em in wheat and barley samples, respectively.

Compared to recent data, the ergot pattern obtained in our study is similar to those reported by other authors. Thus, the EFSA report concluded that the highest average contributions to the total concentration in food samples corresponded to Et (18%), Ecr (15%), Es (12%) and Em (11%) [24]. Indeed, Es, Ekr and Ecr were also reported in another study as predominant in cereal products in Europe [8]; moreover, Es, Econ and Ekr were the most common EA in cereal samples from Luxembourg [16], while Et, Es and Ecr were the most frequently occurring EAs in French cereals [18]. Contrary to our finding, Em and Emn were prevalent in wheat samples from Italy [27].

**Figure 1:**
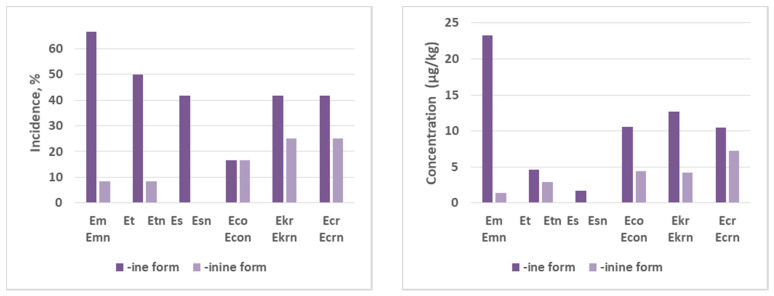
Histograms of incidence and mean concentration of -ine and -inine forms in the positive samples. Abbreviations: ergometrine (Em), ergosine (Es), ergotamine (Et), ergocornine (Eco), ergokryptine (Ekr), ergocristine (Ecr), and their corresponding epimers, ergometrinine (Emn), ergosinine (Esn), ergotaminine (Etn), ergocorninine (Econ), ergokryptinine (Ekrn) and ergocristinine (Ecrn).

The distribution of the -ine and the -inine forms of the EAs was investigated and the results are reported in Figure 1. As shown, the frequency of occurrence of the -ine forms was higher than that of the -inine forms. Indeed, Em, Et and Es were highly predominant in the -ine form, while Eco showed an incidence similar than the -inine form (Econ). Moreover, the mean concentrations for -ine forms was higher in all the cases than the concentrations of the -inine forms. These results are similar to other published studies [8,14,18,24].

### 2.4. Dietary Exposure Estimation

With the purpose to evaluate the risk of exposure of the adult population to EAs through the consumption of barley and wheat, the Probably Dietary Intake (PDI) was calculated for the sum of EAs as indicated in the following equation:

PDI = (C × K)/bw(1)

where C is the mean concentration of the EAs in the sample, K is the average consumption of the food (g/day) and bw is the body weight considered for the adult human population. Once the PDI was calculated, the risk was estimated as the percentage of Tolerable Daily Intake (%TDI), calculated as the ratio of PDI to TDI (µg/kg bw/day) as follows [2,29]:

%TDI = (PDI/TDI) × 100(2)

A tolerable daily intake (TDI) of 0.6 µg/kg bw/day, as proposed by the EFSA CONTAM Panel, has been used as a reference dose [2].

In order to substitute the left-censored data, that is, data below the LOD or LOQ, two exposure scenarios were defined: the lower bound scenario (LB) and the upper bound scenario (UB) [30]. In the lower bound scenario (LB), a zero was assigned when EAs were not detected or were detected below the LOQ. In the upper bound (UB) scenario, the LOD was assigned when EAs were not detected, and the LOQ when EAs were detected at levels below LOQ. The population group considered in this study was adult humans (60 kg) [31]. Consumption data of barley (36 g/day) and wheat (502 g/day) used in this study derived from data reported by the Food and Agriculture Organization (FAO) regarding the nutrition profile for Algerian population [32]. The obtained results are summarized in Table 6. PDI values of EAs through the consumption of barley and wheat were estimated to be 0.003 and 0.074 µg/kg bw/day under the LB scenario. However, when the UB scenario was considered, the PDIs were estimated to be 0.005 and 0.105 µg/kg bw/day for barley and wheat, respectively. These PDI values obtained for adult population were below the proposed TDI (0.6 µg/kg bw/day for the sum of 12 EAs), representing less than 17.6% of TDI in both the LB and UB approaches.

This finding suggests that barley and wheat from Algeria could be considered safe for the average adult consumers concerning EAs. However, there are other population groups, such as infants or groups with higher intake of these cereals, which could suffer a higher risk when consuming these products. As a consequence, monitoring programs to control the presence of EAs are required to ensure protection of all consumers. 

## 3. Conclusions

The present study reported the first data about the presence of 12 EAs in cereals (barley and wheat) from Algeria. Wheat showed a higher incidence than barley (26.7% and 13.3%, respectively), and the results of our study revealed low contamination of EAs in barley (range for the sum EAs of 17.8–53.9 µg/kg) and wheat (range for the sum EAs of 3.66–76.0 µg/kg). The study shows variability in the pattern of EAs among the positive samples, the most frequent being Es, Ekr and Ecr in wheat, and Em in barley, and emphasizes the importance of including the six EAs and their epimers in the risk assessment. In view of the results, there is no evidence of risk linked with the EAs intake through the consumption of barley and wheat in Algeria since the levels of the PDI obtained from the studied samples are far below the TDI proposed by the EFSA. Nevertheless, this study includes a limited number of samples and locations, and considering that EA production depends on climate conditions, it would be advisable to collect more data on the incidence of EAs in cereal samples from different locations and in different seasons. 

## 4. Materials and Methods

### 4.1. Reagents and Materials

Ultrapure water was obtained from a Milli-Q Plus system (Millipore Bedford, MA, USA). LC-MS grade methanol (MeOH), acetonitrile (MeCN) and ammonium carbonate ((NH_4_)_2_CO_3_) were purchased from VWR International (Barcelona, Spain), and formic acid eluent additive for LC–MS from Sigma Aldrich (St. Louis, MO, USA). Z-Sep+ sorbent was supplied by Supelco (Bellefonte, PA, USA), and C18 and PSA sorbents by Agilent Technologies (Madrid, Spain).

Fine film dried standards of Em, Emn, Et and Etn were purchased from Romer labs (Getzersdorf, Austria), and the rest (Es, Esn, Eco, Econ, Ekr and Ekrn) was obtained from Techno Spec (Barcelona, Spain). The standards were, as indicated by the manufacturer, reconstituted in 5 mL of solvent (MeCN), to give certified concentrations of 500 µg/mL and of 125 µg/mL for the -ine and -inine isomers, respectively. To prevent rapid epimerization of EAs in the solution, defined volumes of freshly prepared individual or mixed standard solutions were pipetted into amber glass tubes, evaporated to dryness under a stream of nitrogen, and kept at −20 °C. These frozen standards were reconstituted in the proper volume of solvent immediately before use.

A universal 320R centrifuge (Hettich ZENtrifugen, Tuttlingen, Germany), a vortex-2 Genie (Scientific Industries, Bohemia, NY, USA), an evaporator System (System EVA-EC, from VLM GmbH, Bielefeld, Germany) and a high-speed solid crusher (Hukoer, China) were used to process samples.

Nylon syringe filters (13 mm, 0.22 μm, from VWR) were used for filtration of extracts prior to their injection into the UHPLC-MS/MS system.

### 4.2. Samples

A total of 60 samples (30 samples of barley and 30 of wheat) destined for human consumption were randomly collected during the year 2018 from retail shops and supermarkets from three cities in Algeria: Aint Temouchent (10 samples of barley and 10 of wheat), Oran (10 samples of barley and 10 of wheat) and Tiaret (10 samples of barley and 10 of wheat). No information about the country of production was available for the samples. The sampling was done according to Malysheva et al. and European Commission Regulation [8,33]. Briefly, from each sample lot (bag of 50 kg) of barley and wheat, five sub-samples of 200 g were taken from different positions (from the top, middle and bottom of the bag) and thoroughly mixed to achieve a 1-kg aggregate sample. From the aggregate sample, a laboratory sample of 200 g was taken, grinded, homogenized and stored at −20 °C until analysis.

### 4.3. Sample Preparation

A previously described sample treatment was adapted with some modifications [34]. Briefly, 1 g of homogenized sample was placed in a 50-mL falcon tube, 4 mL of MeCN and ammonium carbonate 5 mM (85:15, *v*/*v*) were added and the mixture was vortexed (1 min) and centrifuged (5 min, 9000 rpm, 4 °C). Then, the whole supernatant was transferred to a 15-mL falcon tube containing 150 mg of a mixture of C18/Z-Sep^+^ (50/50) dispersive sorbents for cleaning-up. Next, the mixture was vortexed (1 min) and centrifuged (5 min, 9000 rpm, 4 °C). The supernatant was transferred to a 4-mL vial, evaporated to dryness with nitrogen and reconstituted to a final volume of 750 µL with MeOH:water (50:50, *v*/*v*). Finally, the samples were filtered through a 0.22 µm nylon filter before their injection and the 12 EAs were determined by UHPLC-MS/MS. 

### 4.4. UHPLC-MS/MS Analysis

Separation of EAs was carried out in an Agilent 1290 Infinity LC (Agilent Technologies, Waldbronn, Germany), while detection and quantification were performed in an API 3200 triple quadrupole mass spectrometer (ABSciex, Darmstadt, Germany) with electrospray ionization (ESI) and Analyst version 1.6.3 software. An Agilent Zorbax Eclipse Plus RRHD C18 (50 × 2.1 mm, 1.8 µm) at 35 °C was used as chromatographic column. The mobile phase consisted of a mixture of water (A) and MeOH (B), both containing 0.3% formic acid. The gradient elution program was as follows: 0 min: 30% B; 6 min: 60% B; 9 min: 60% B; 10 min: 30 B; 12 min: 30% B. The flow rate was 0.4 mL/min and the injection volume was 5 µL.

The mass spectrometer operated in the positive electrospray ionization mode (ESI+) under multiple reaction monitoring (MRM) conditions (detailed conditions are included in the Supplementary Materials, Table S2). The ionization source parameters were set as follows: temperature of the source 500 °C; collision gas (nitrogen) 5 psi; voltage of the ion spray 5 kV; curtain gas (nitrogen) 30 psi; nebulizing gas (GAS 1) and drying gas (GAS 2), both of them nitrogen set at 50 psi. The monitored ions were the protonated molecules [M + H]^+^, except for Esn, Etn, Econ, Ecrn and Ekrn, where the signal at *m*/*z* corresponding to [M − H_2_O + H]^+^ was higher than that of the protonated molecules, in accordance with previous works [1].

### 4.5. Method Validation

Validation of the method was assessed for each cereal by studying linearity, LOD, LOQ, matrix effect, trueness (in terms of recovery), and precision for each EA. Spiked blank samples (previously analyzed to confirm a negative result) were used.

Procedural calibration curves were prepared by spiking blank samples at six concentration levels (processed in duplicate) within the analytical range from 2 to 100 μg/kg. Linearity was evaluated with the determination coefficient (R^2^). LOD and LOQ were defined as 3 × S/N and 10 × S/N, respectively.

The matrix effect (ME%) for each analyte was evaluated preparing blank samples and spiking the extracts just before analysis, at two concentration levels (5 and 50 μg/kg), and was calculated according to the following equation:

ME% = 100 × (signal of spiked extract–signal of standard solution) / signal of standard solution.(3)

Recovery studies were carried out by fortifying blank samples at two concentration levels (5 and 50 μg/kg). Each sample was processed in triplicate and injected three times. The peak area ratios of the sample spiked before extraction to sample spiked after extraction were used to calculate the extraction recovery.

Precision was estimated at two concentration levels (5 and 50 μg/kg) and expressed as the relative standard deviation (%RSD) of the results obtained from three samples injected in triplicate on the same day (intra-day precision) and on three different days (inter-day precision). 

## Figures and Tables

**Table 1:** Linear regression equations, linearity (expressed as determination coefficient, R^2^), limits of detection (LOD) and limits of quantification (LOQ) of the UHPLC-MS/MS determination of EAs for barley and wheat.

	Barley				Wheat			
EA	Linear Regression Equation	R^2^	LOD (µg/kg)	LOQ (µg/kg)	Linear Regression Equation	R^2^	LOD (µg/kg)	LOQ (µg/kg)
Em	y = 86.47x + 285.1	0.997	1.18	3.92	y = 108.1x + 364.3	0.992	1.00	3.33
Emn	y = 4352x + 482.4	0.997	0.12	0.50	y = 6607x − 336.0	0.993	0.15	0.49
Es	y = 1042x − 181.7	0.997	0.33	1.09	y = 1321x + 127.4	0.998	0.15	0.50
Esn	y = 1293x + 931.4	0.998	0.21	0.71	y = 1783x + 789.8	0.998	0.18	0.59
Et	y = 739.9x − 491.1	0.996	0.37	1.22	y = 768.2x − 288.6	0.996	0.29	0.97
Etn	y = 949.1x + 2036	0.995	0.32	1.06	y = 1169x + 917	0.996	0.30	1.00
Eco	y = 967.3x + 1534	0.995	0.35	1.16	y = 1294x + 324.5	0.998	0.26	0.86
Econ	y = 772.7x + 230.3	0.998	0.40	1.35	y = 1032x − 205.7	0.998	0.32	1.06
Ekr	y = 850.2x − 42.54	0.998	0.27	0.91	y = 1084x − 797.9	0.997	0.33	1.09
Ekrn	y = 1676x + 1589	0.996	0.21	0.71	y = 2323x − 611.3	0.998	0.20	0.68
Ecr	y = 605.4x + 490.6	0.996	0.45	1.52	y = 796.8x − 414.5	0.996	0.32	1.08
Ecrn	y = 730.1x + 1038	0.996	0.29	0.97	y = 1028.5x − 709.4	0.998	0.32	1.05

Abbreviations: ergometrine (Em), ergosine (Es), ergotamine (Et), ergocornine (Eco), ergokryptine (Ekr), ergocristine (Ecr), and their corresponding epimers, ergometrinine (Emn), ergosinine (Esn), ergotaminine (Etn), ergocorninine (Econ), ergokryptinine (Ekrn) and ergocristinine (Ecrn).

**Table 2:** Matrix effect and recovery of the UHPLC-MS/MS determination of EAs for barley and wheat.

	Barley				Wheat			
	Matrix Effect (%)		Recovery (%)		Matrix Effect (%)		Recovery (%)	
EA	5 μg/kg	50 μg/kg	5 μg/kg	50 μg/kg	5 μg/kg	50 μg/kg	5 μg/kg	50 μg/kg
Em	−51.7	−43.4	99.0	89.3	−38.8	−26.2	84.9	86.0
Emn	−38.5	−30.5	86.6	88.9	−25.8	−20.1	92.6	89.3
Es	−8.4	−10.7	94.8	96.9	−11.4	−7.8	105	103
Esn	−8.6	−6.6	101	101	−15.9	−13.6	99.8	104
Et	−14.5	−9.9	105	103	−13.4	−8.7	102	91.6
Etn	−10.3	−4.9	100	104	−12.3	−8.2	107	104
Eco	−12.7	−15.7	98.8	99.4	−11.8	−6.9	109	99.5
Econ	−1.9	−7.9	104	102	−8.6	−6.7	106	94.6
Ekr	−11.0	−11.3	94.6	95.8	−10.3	−10.7	93.7	92.1
Ekrn	−8.5	−4.7	98.0	97.8	−13.2	−7.4	98.0	95.5
Ecr	−12.6	−4.4	95.8	96.6	−12.4	−8.5	98.4	90.7
Ecrn	−5.7	−4.6	97.7	97.4	−10.8	−6.7	93.3	92.2

Abbreviations: ergometrine (Em), ergosine (Es), ergotamine (Et), ergocornine (Eco), ergokryptine (Ekr), ergocristine (Ecr), and their corresponding epimers, ergometrinine (Emn), ergosinine (Esn), ergotaminine (Etn), ergocorninine (Econ), ergokryptinine (Ekrn) and ergocristinine (Ecrn).

**Table 3:** Precision study (%RSD) of the UHPLC-MS/MS determination of EAs for barley and wheat.

	Barley				Wheat			
	Intra-Day Precision (n = 9)		Inter-Day Precision (n = 9)		Intra-Day Precision (n = 9)		Inter-Day Precision (n = 9)	
EA	5 μg/kg	50 μg/kg	5 μg/kg	50 μg/kg	5 μg/kg	50 μg/kg	5 μg/kg	50 μg/kg
Em	6.5	3.8	5.6	6.4	9.0	5.9	11	11
Emn	4.1	2.4	9.4	6.2	3.6	5.4	4.5	6.3
Es	4.9	6.2	9.4	7.7	9.2	6.3	8.3	7.4
Esn	6.0	3.0	7.3	9.2	5.2	7.5	6.0	9.6
Et	4.6	6.9	8.7	6.0	5.9	7.6	7.3	10
Etn	6.3	4.5	9.2	6.5	9.3	7.6	11	7.7
Eco	6.8	4.4	9.6	5.2	7.8	5.0	8.3	6.6
Econ	5.5	6.1	6.4	8.0	7.4	4.6	8.5	5.8
Ekr	4.8	4.3	9.0	4.9	9.6	5.6	11	7.4
Ekrn	3.0	4.8	5.6	6.8	6.6	4.6	10	10
Ecr	6.6	4.6	6.2	4.7	8.2	6.9	10	7.5
Ecrn	4.2	3.6	6.1	4.5	6.0	4.6	7.3	5.6

Abbreviations: ergometrine (Em), ergosine (Es), ergotamine (Et), ergocornine (Eco), ergokryptine (Ekr), ergocristine (Ecr), and their corresponding epimers, ergometrinine (Emn), ergosinine (Esn), ergotaminine (Etn), ergocorninine (Econ), ergokryptinine (Ekrn) and ergocristinine (Ecrn).

**Table 4:** Summary of the EA occurrence in barley and wheat samples.

Sample	I ^a^ (%)	Mean ^b^ (µg/kg)	Range ^c^ (µg/kg)	Distribution (µg/kg)	
				<10	10–100
Barley (n = 30)	4 (13.3%)	35.4	17.8–53.9	0	4
Wheat (n = 30)	8 (26.7%)	33.1	3.66–76.0	1	7
Total (n = 60)	12 (20%)	34.3	3.66–76.0	1	11

^a^ Incidence of positive samples (percentage %); ^b^ Mean value for positive samples; ^c^ minimum concentration value–maximum concentration value.

**Table 5:** Summary of individual ergot alkaloid concentrations in positive samples.

	Parameter		Ergot Alkaloid										
		Em	Emn	Es	Esn	Et	Etn	Eco	Econ	Ekr	Ekrn	Ecr	Ecrn
Wheat (n = 8)	I ^a^ (%)	4 (50%)	1 (12.5%)	5 (62.5%)	0	3 (37.5%)	1 (12.5%)	2 (25%)	2 (25%)	5 (62.5%)	3 (37.5%)	5 (62.5%)	3 (37.5%)
	Mean ^b^ (µg/kg)	13.5	1.42	1.70	-	6.2	2.91	10.5	4.4	12.7	4.2	10.4	7.2
	LOD-LOQ ^c^	2 (25%)	3 (37.5%)	0	3 (37.5%)	0	0	1 (12.5%)	1 (12.5%)	0	2 (25%)	0	1 (12.5%)
	Min ^d^ (µg/kg)	3.52	1.42	0.62	-	1.15	2.91	8.68	3.84	1.56	3.28	2.10	1.50
	Max ^e^ (µg/kg)	24.9	1.42	3.30	-	13.6	2.91	12.40	4.90	26.2	5.88	28.6	12.2
Barley (n = 4)	I ^a^ (%)	4 (100%)	0	0	0	3 (75%)	0	0	0	0	0	0	0
	Mean ^b^ (µg/kg)	33.1	-	-	-	3.01	-	-	-	-	-	-	-
	LOD-LOQ ^c^	0	4	0	0	0	0	0	0	0	0	0	0
	Min ^d^ (µg/kg)	17.8	-	-	-	2.34	-	-	-	-	-	-	-
	Max ^e^ (µg/kg)	50.0	-	-	-	3.90	-	-	-	-	-	-	-

^a^ Incidence of samples ≥LOQ; ^b^ Mean value for samples ≥LOQ; ^c^ Incidence of samples with concentration ≥LOD and ≤LOQ; ^d^ minimum concentration value; ^e^ maximum concentration value. Abbreviations: ergometrine (Em), ergosine (Es), ergotamine (Et), ergocornine (Eco), ergokryptine (Ekr), ergocristine (Ecr), and their corresponding epimers, ergometrinine (Emn), ergosinine (Esn), ergotaminine (Etn), ergocorninine (Econ), ergokryptinine (Ekrn) and ergocristinine (Ecrn).

**Table 6:** Dietary exposure to EAs through the consumption of barley and wheat (PDI: Probably Dietary Intake; TDI: Tolerable Daily Intake).

Samples	Mean (µg/kg)		PDI (µg/kg bw/day)		TDI %	
	LB	UB	LB	UB	LB	UB
Wheat (n = 30)	8.83	12.59	0.074	0.105	12.32	17.56
Barley (n = 30)	4.71	8.68	0.003	0.005	0.47	0.87

## Supplementary Materials

The following are available online at https://www.mdpi.com/article/10.3390/toxins13050316/s1, Table S1. Results of positive samples (concentration of EA higher than LOD, n = 9). <LOQ: concentration below the LOQ for the EA (detected but not quantified, concentration considered as equal to zero); Table S2. MS parameters for the different target analytes studied in the proposed UHPLC-MS/MS method. Figure S1: Chemical structures of the studied ergot alkaloids.

## References

[1] Di Mavungu D., Malysheva S.V., Sanders M., Larionova D., Robbens J., Dubruel P., Van Peteghem C., De Saeger S. (2012). Development and validation of a new LC-MS/MS method for the simultaneous determination of six major ergot alkaloids and their corresponding epimers. Application to some food and feed commodities. Food Chem..

[2] EFSA Panel on Contaminants in the Food Chain (CONTAM) (2012). Scientific opinion on ergot alkaloids in food and feed. EFSA J..

[3] Mulder P.P.J., Pereboom-de Fauw D.P.K.H., Hoogenboom R.L.A.P., de Stoppelaar J., de Nijs M. (2015). Tropane and ergot alkaloids in grain-based products for infants and young children in the Netherlands in 2011–2014. Food Addit. Contam. Part B Surveill..

[4] Krskaab R., Crews C. (2008). Significance, chemistry and determination of ergot alkaloids: A review. Food Add. Contam..

[5] De Costa C. (2002). St Anthony’s fire and living ligatures: A short history of ergometrine. Lancet.

[6] Krska R., Stubbings G., MacArthur R., Crews C. (2008). Simultaneous determination of six major ergot alkaloids and their epimers in cereals and foodstuffs by LC-MS-MS. Anal. Bioanal. Chem..

[7] Scott P.M. (2009). Ergot alkaloids: Extent of human and animal exposure. World Mycotoxin J..

[8] Malysheva S.V., Larionova D.A., Diana Di Mavungu J., De Saeger S. (2014). Pattern and distribution of ergot alkaloids in cereals and cereal products from European countries. World Mycotoxin J..

[9] Crews C., Anderson W.A.C., Rees G., Krska R. (2009). Ergot alkaloids in some rye-based UK cereal products. Food Addit. Contam. Part B Surveill..

[10] Paterson R.R.M., Lima N. (2011). Further mycotoxin effects from climate change. Food Res. Int..

[11] European Commission (2012). Commission Recommendation of 15 March 2012 on the monitoring of the presence of ergot alkaloids in feed and food. Off. J. Eur. Union.

[12] European Commission (2006). Commission Regulation (EC) No 1881/2006 of 19 December 2006 setting maximum levels for certain contaminants in foodstuffs. Off. J. Eur. Union.

[13] European Commission (2002). Directive 2002/32/EC of the European Parliament and of the Council of 7 May 2002 on undesirable substances in animal feed. Off. J. Eur. Communities.

[14] Tittlemier S.A., Drul D., Roscoe M., McKendry T. (2015). 2015. Occurrence of ergot and ergot alkaloids in western Canadian wheat and other Cereals. J. Agric. Food Chem..

[15] Holderied I., Rychlik M., Elsinghorst P.W. (2019). Optimized analysis of ergot alkaloids in rye products by liquid chromatography-fluorescence detection applying lysergic acid diethylamide as an internal standard. Toxins.

[16] Schummer C., Brune L., Moris G. (2018). Development of a UHPLC-FLD method for the analysis of ergot alkaloids and application to different types of cereals from Luxembourg. Mycotoxin Res..

[17] Arroyo-Manzanares N., Gámiz-Gracia L., García-Campaña A.M., Diana di Magunvu J., De Saeger S., Mérillon J.M., Ramawat K. (2016). Ergot alkaloids: Chemistry, biosynthesis, bioactivity, and methods of analysis. Fungal Metabolites.

[18] Orlando B., Maumené C., Piraux F. (2017). Ergot and ergot alkaloids in French cereals: Occurrence, pattern and agronomic practices for managing the risk. World Mycotoxin J..

[19] Crews C. (2015). Analysis of ergot alkaloids. Toxins.

[20] Tantaoui-Elaraki A., Riba A., Oueslati S., Zinedine A. (2018). Toxigenic fungi and mycotoxin occurrence and prevention in food and feed in northern Africa–a review. World Mycotoxin J..

[21] Mahdjoubi C.K., Arroyo-Manzanares N., Hamini-Kadar N., García-Campaña A.M., Mebrouk K., Gámiz-Gracia L. (2020). Multi-mycotoxin occurrence and exposure assessment approach in foodstuffs from Algeria. Toxins.

[22] European Commission (2002). Commission Decision of 12 August 2002 implementing Council Directive 96/23/EC concerning the performance of analytical methods and the interpretation of results (2002/657/EC). Off. J. Eur. Commun..

[23] SANTE/12089/2016 Guidance Document on Identification of Mycotoxins in Food and Feed. Implemented by 01 January 2017. https://ec.europa.eu/food/sites/food/files/safety/docs/cs_contaminants_sampling_guid-doc-ident-mycotoxins.pdf.

[24] Arcella D., Gómez-Ruiz J.A., Innocenti M.L., Roldán R. (2017). Scientific Report: Human and animal dietary exposure to ergot alkaloids. EFSA J..

[25] Uhlig S., Eriksen G.S., Hofgaard I.S., Krska R., Beltrán E., Sulyok M. (2013). Faces of a changing climate: Semi-quantitative multi-mycotoxin analysis of grain grown in exceptional climatic conditions in Norway. Toxins.

[26] Guo Q., Shao B., Du Z., Zhang J. (2016). Simultaneous determination of 25 ergot alkaloids in cereal samples by ultraperformance liquid chromatography-tandem mass spectrometry. J. Agric. Food Chem..

[27] Debegnach F., Patriarca S., Brera C., Gregori E., Sonego E., Moracci G., De Santis B. (2019). Ergot alkaloids in wheat and rye derived products in Italy. Foods.

[28] Arroyo-Manzanares N., De Ruyck K., Uka V., Gámiz-Gracia L., García-Campaña A.M., De Saeger S., Diana Di Mavungu J. (2018). In-house validation of a rapid and efficient procedure for simultaneous determination of ergot alkaloids and other mycotoxins in wheat and maize. Anal. Bioanal. Chem..

[29] Rodríguez-Carrasco Y., Ruiz M.J., Font G., Berrada H. (2013). Exposure estimates to *Fusarium* mycotoxins through cereals intake. Chemosphere.

[30] EFSA (2010). Management of left-censored data in dietary exposure assessment ofchemical substances. EFSA J..

[31] International Programme on Chemical Safety (IPCS) (2009). Principles and Methods for the Risk Assessment of Chemicals in Food. Environmental Health Criteria 240.

[32] FAO Statistics Division (FAOSTAT) Food and Agricultural Commodities Production. http://faostat.fao.org/site/339/default.aspx.

[33] European Commission (2006). Commission regulation (EC) No 401/2006 of 23 February 2006 laying down the methods of sampling and analysis for the official control of the levels of mycotoxins in foodstuffs. Off. J. Eur. Union.

[34] Arroyo-Manzanares N., Rodríguez-Estévez V., García-Campaña A.M., Castellón-Rendón E., Gámiz-Gracia L. (2021). Determination of principal ergot alkaloids in swine feeding. J. Sci. Food Agric..

