# Supplementary material for: Occurrence of Ergot Alkaloids in Barley and Wheat from Algeria
Source: Toxins (Basel). 2021 Apr 28;13(5):316. doi: 10.3390/toxins13050316 (PMC8145663; doi:10.3390/toxins13050316)
Supplement: Supplementary file 1 [file toxins-13-00316-s001.zip › toxins-1185837-supplementary.pdf]

Occurrence of Ergot Alkaloids in Barley and Wheat from Algeria

Laura Carbonell-Rozas, Choukri Khelifa Mahdjoubi, Natalia Arroyo-Manzanares, Ana M. García-Campaña and Laura Gámiz-Gracia

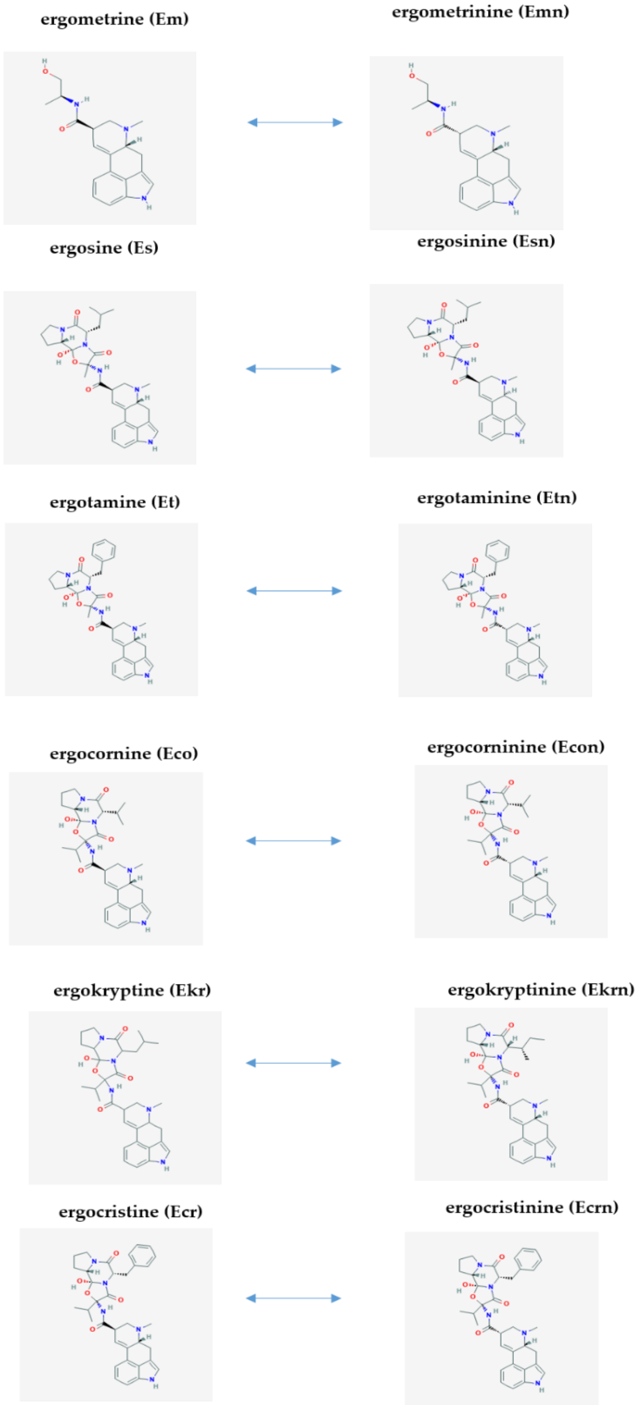

Figure S1. Chemical structures of the studied ergot alkaloids.

Table S1. Results of positive samples (concentration of EA higher than LOD, n=9). <LOQ: concentration below the LOQ for the EA (detected but not quantified, concentration considered as equal to zero).

BARLEY			
**Sample***	**Ergot Alkaloid**	**Concentration (µg/kg)**	**%RSD**
T1	Em	30.4	2.2
	Emn	<LOQ	---
	Et	2.8	0.4
	**Total EAs (T1)**	**33.2**	
T3	Em	50.0	2.5
	Emn	<LOQ	---
	Et	3.9	0.5
	**Total EAs (T3)**	**53.9**	
T7	Em	34.2	2.2
	Emn	<LOQ	---
	Et	2.34	0.3
	**Total EAs (T7)**	**36.5**	
T10	Em	17.8	1.4
	Emn	<LOQ	---
	**Total EAs (T10)**	**17.8**	

Table S1. (cont.). Results of positive samples (concentration of EA higher than LOD, n=9). <LOQ: concentration below the LOQ for the EA (detected but not quantified, concentration considered as equal to zero).

WHEAT			
**Sample***	**Ergot Alkaloid**	**Concentration (µg/kg)**	**%RSD**
O2	Em	13.6	1.3
	**Total EAs (O2)**	**13.6**	
T8	Em	24.9	1.2
	Emn	1.42	0.2
	Es	0.62	0.1
	Et	3.71	0.6
	Eco	<LOQ	---
	Econ	<LOQ	---
	Ekr	17.7	1.9
	Ekrn	3.45	0.3
	Ecr	16.1	1.0
	Ecrn	8.03	0.2
	**Total EAs (T8)**	**76.0**	
O7	Es	1.66	0.9
	Esn	<LOQ	---
	Eco	8.68	1.3
	Econ	3.84	1.2
	Ekr	13.2	0.4
	Ekrn	3.28	1.0
	**Total EAs (O7)**	**30.7**	
AT1	Em	<LOQ	---
	Emn	<LOQ	---

	Es	1.61	0.4
	Esn	<LOQ	---
	Et	1.15	0.3
	Ekr	4.76	0.8
	Ekrn	<LOQ	---
	Ecr	2.54	0.3
	**Total EAs (AT1)**	**10.1**	
T9	Em	<LOQ	---
	Ekr	1.56	0.2
	Ekrn	<LOQ	---
	Ecr	2.10	0.3
	Ecrn	<LOQ	---
	**Total EAs (T9)**	**3.66**	
O4	Em	11.78	1.5
	Emn	<LOQ	---
	Es	1.30	0.2
	Ecr	2.75	0.3
	Ecrn	1.50	0.1
	**Total EAs (O4)**	**17.3**	
T10	Em	3.52	0.8
	Emn	<LOQ	---
	Et	13.6	1.3
	Etn	2.91	0.7
	Ecr	28.6	1.0
	Ecrn	12.2	0.5
	**Total EAs (T10)**	**60.8**	
	Es	3.3	0.9
	Esn	<LOQ	---
	Eco	12.4	1.5
	Econ	4.9	0.8
	Ekr	26.2	0.9
	Ekrn	5.88	1.6
	**Total EAs (O10)**	**52.7**	

*Sample code: A: samples from Aint Temouchent; O: samples from Oran; T: samples from Tiaret.

Table S2. MS parameters for the different ergot alkaloids studied in the proposed UHPLC-MS/MS method. .

	Retention time (min)	Precursor ion (m/z)	Molecular ion	DP[a]	EP[a]	Product ions[b]	CE[a]	CXP[a]
**Em**	0.49	326.0	$[M+H]^+$	51	5.0	223.0 (Q)	23	4.0
						208.1 (I)	37	4.0
**Emn**	0.80	326.0	$[M+H]^+$	46	6.0	208.1 (Q)	39	6.0
						223.1 (I)	33	6.0
**Es**	3.82	548.2	$[M+H]^+$	61	5.0	208.2 (Q)	57	4.0
						223.1 (I)	45	8.0
**Esn**	3.56	530.2	$[M-H_2O+H]^+$	66	6.5	223.2 (Q)	37	6.0
						263.1 (I)	33	6.0
**Et**	4.24	582.2	$[M+H]^+$	56	7.0	208.2 (Q)	55	4.0
						223.2 (I)	45	4.0
**Etn**	4.08	564.2	$[M-H_2O+H]^+$	66	6.0	223.0 (Q)	41	6.0
						297.1 (I)	33	6.0
**Eco**	4.18	562.2	$[M+H]^+$	46	4.5	268.1 (Q)	33	6.0
						208.2 (I)	55	4.0
**Econ**	5.19	544.2	$[M-H_2O+H]^+$	61	8.5	277.1 (Q)	31	6.0
						223.1 (I)	37	6.0
**Ekr**	5.24	576.2	$[M+H]^+$	86	6.0	208.3 (Q)	59	6.0
						268.1 (I)	31	6.0
**Ekrn**	5.85	576.2	$[M-H_2O+H]^+$	36	7.0	223.0 (Q)	45	6.0
						558.0 (I)	21	6.0
**Ecr**	5.44	610.2	$[M+H]^+$	56	6.5	268.2 (Q)	35	6.0
						208.1 (I)	57	6.0
**Ecrn**	6.08	592.2	$[M-H_2O+H]^+$	71	7.5	305.1 (Q)	33	4.0
						223.2 (I)	39	6.0

[a] Declustering potential (DP), Entrance potential (EP), Collision Cell Exit Potential (CXP) and Collision Energy (CE). All expressed in voltage. [b] Product ions: (Q) Transition used for quantification, (I) Transition employed to confirm the identification.
